# Health Effects of Calcium: Evidence From Mendelian Randomization Studies

**DOI:** 10.1002/jbm4.10542

**Published:** 2021-08-31

**Authors:** Yiheng Chen, Vincenzo Forgetta, J. Brent Richards, Sirui Zhou

**Affiliations:** ^1^ Department of Human Genetics McGill University Montréal QC Canada; ^2^ Lady Davis Institute, Jewish General Hospital McGill University Montréal QC Canada; ^3^ Department of Epidemiology, Biostatistics and Occupational Health McGill University Montréal QC Canada; ^4^ Department of Twin Research King's College London London UK

**Keywords:** CALCIUM, CANCER, CARDIOVASCULAR DISEASE, MENDELIAN RANDOMIZATION, MUSCULOSKELETAL DISEASES

## Abstract

Calcium is widely used in conjunction with vitamin D to prevent osteoporosis. The use of calcium supplementation is also promoted for its potential benefits in lowering the risk for metabolic syndromes and cancers. However, the causal link between calcium and various health outcomes remains unclear. This review focuses on the evidence from 24 Mendelian randomization (MR) studies that were designed to minimize bias from confounding and reverse causation. These MR studies evaluated the effect of lifelong genetically higher serum calcium levels on various health outcomes. Overall, available MR studies found no conclusive effects of serum calcium levels on bone mineral density and fracture, ischemic stroke and heart failure, cancers, type 2 diabetes, Parkinson disease, or offspring birth weight. However, a higher serum calcium concentration was reported to have estimated causal effects on increased risks for coronary artery disease (especially myocardial infarction), migraine, renal colic, allergy/adverse effect of penicillin, and reduced risks for osteoarthrosis and osteoarthritis. In conclusion, supplementation of calcium in individuals from the general population is not predicted to influence the risk of most investigated diseases to date. Moreover, long‐term high serum calcium concentrations may result in adverse health outcomes. © 2021 The Authors. *JBMR Plus* published by Wiley Periodicals LLC on behalf of American Society for Bone and Mineral Research.

## Introduction

As the most abundant mineral in human body, 99% of calcium is used by the skeletal system while the remaining 1% circulates between the extracellular fluid and intracellular stores and participates in essential biological activities such as constituting the signaling transduction system and mediating cellular functions.^(^ ^1^ ^)^ Of the calcium circulating in the blood, about half is bound to proteins while the other half is in a bioactive ionized form.^(^ ^2^ ^)^ Blood calcium level is tightly regulated, with a normal range of serum calcium concentration between 8.6 and 10.3 mg/dL or 2.2 to 2.6 mmol/L*. Major* hormones participating in the regulation of calcium homeostasis are parathyroid hormone (PTH) and 1,25‐dihydroxyvitamin D (1,25(OH)_2_D). They work through their corresponding receptors in different organs such as bone, the gastrointestinal tract, and the kidney to maintain serum calcium levels.^(^ ^3^ ^)^ The regulation of circulating calcium also involves many proteins. For example, the calcium‐sensing receptor detects extracellular levels of calcium ions and can signal the release of PTH, whereas 25‐hydroxyvitamin D‐24‐hydroxylase can influence calcium level by controlling the degradation of serum 1,25(OH)_2_D.^(^ ^4^, ^5^ ^)^

Many studies have aimed to investigate the role of calcium and vitamin D deficiency on nutritional rickets^(^ ^6^ ^)^ and osteoporosis^(^ ^7^ ^)^; however, the effect of calcium supplementation alone on skeletal diseases is still in debate.^(^ ^8^ ^)^ Additionally, studies have observed the association between higher calcium intake and lower risks for other diseases such as metabolic disorders and cancers.^(^ ^9^ ^)^ Based on these findings, calcium supplementation is promoted by many health authorities to reduce the risk of osteoporosis and fractures^(^ ^10^, ^11^ ^)^ and protect against colorectal and breast cancer.^(^ ^12^ ^)^ However, studies also found that increased serum calcium, including short‐term use of calcium supplements, is associated with an increased risk of cardiovascular disease and mortality.^(^ ^13^, ^14^ ^)^ Therefore, an important clinical question is whether calcium has any causal effect on diseases (eg, osteoporosis, cancer, and cardiovascular diseases). In particular, does increased serum calcium level provide any demonstrable health benefits?

The majority of studies investigating the effect of serum calcium status or calcium supplements on health outcomes are traditional observational studies. However, these findings can be influenced by selection bias, recall bias, and confounding from unmeasured variables (ie, residual confounding). This is particularly problematic for modifiable risk factors such as calcium status. For example, recall bias and residual confounding may happen when dietary and supplementation information is collected to estimate the level of calcium intake. Moreover, reverse causation could occur in observational studies when disease outcome influences the level of exposure. This is a particular concern for calcium‐related diseases such as cancers, because the disease onset may occur before the diagnosis and assessment of calcium status.^(^ ^15^ ^)^ Another widely used study design is the randomized controlled trial (RCT). It can overcome many of the aforementioned biases and has been used to investigate the causal effect of calcium supplementation on diseases. However, there are few high‐quality RCTs in the field of calcium research. Many existing calcium RCTs have relatively small sample sizes or specific demographic profiles (eg, populations with certain cultural and physical environment, calcium status, or age category), and thus have limited generalizability. Besides, RCTs usually have a short follow‐up timeframe whereas complex diseases can take years to be diagnosed and/or influenced by calcium exposure. Last, the high cost of RCTs can be prohibitive, especially when interventions consist of off‐patent supplementation or dietary modification.

Owing to the establishment of large biobanks and reduced cost of genotyping, many well‐powered genomewide association studies (GWASs) have been conducted recently. The summary statistics from these GWASs have contributed to the emergence of Mendelian randomization (MR) studies, a genetic epidemiological approach that can be used to estimate causal relationships between exposures and outcomes, but with strong assumptions. MR is a helpful complement for high‐quality RCTs in studying casual relationships because it overcomes some of the limitations of observational studies. This review discusses the use of MR in calcium studies and summarizes the recent MR studies that investigate the effect of serum calcium levels on various health outcomes.

### MR and genetically predicted calcium level

**Fig. 1:**
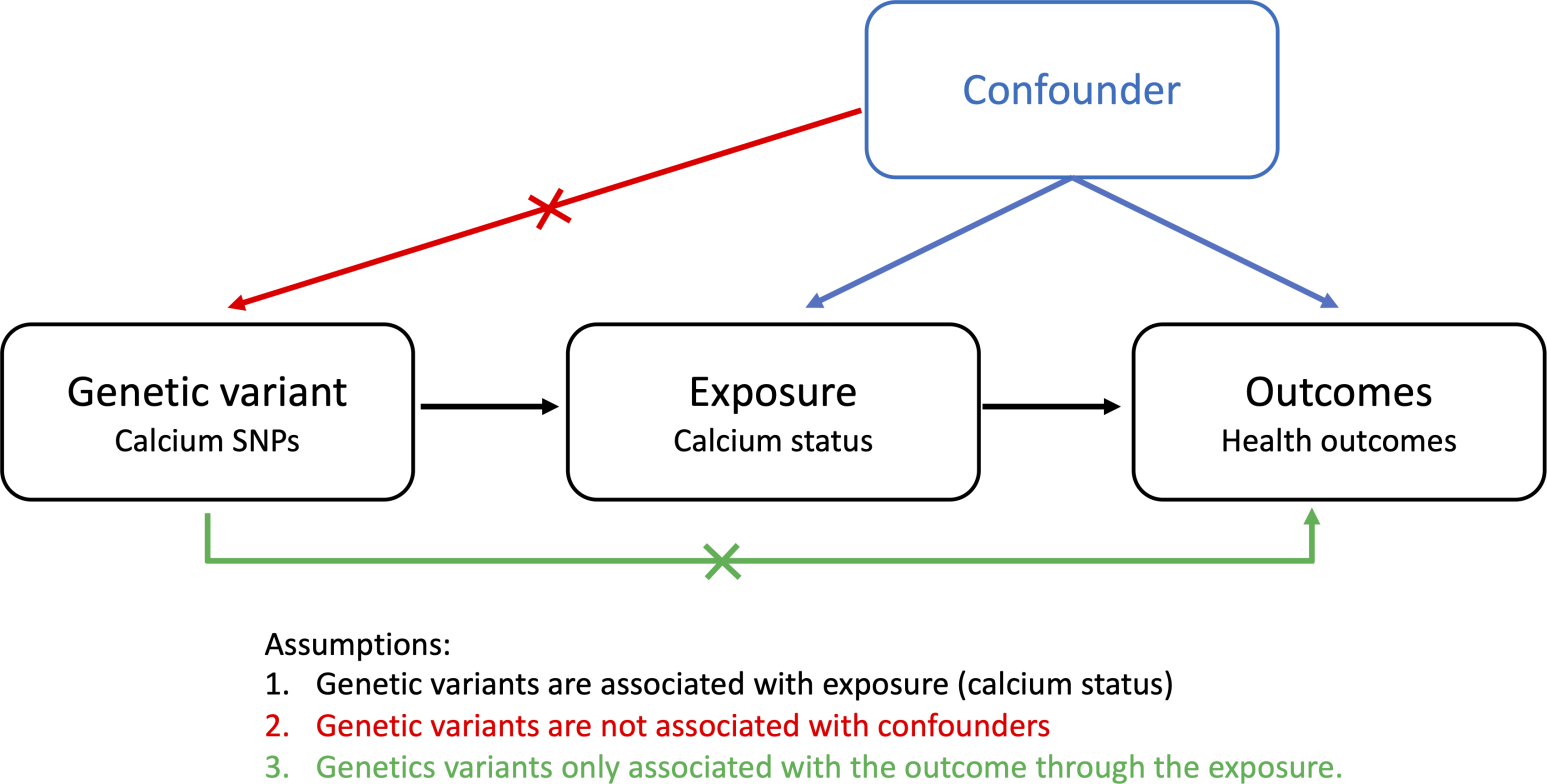
Schematic representation of MR analyses and assumptions. The causal effect of calcium on the risk of various diseases and traits can be estimated using the genetic variants associated with the exposure and outcome.

MR uses genetic variants as naturally randomized instrumental variables to test the causal association between risk factors (exposures) and outcomes. If a modifiable risk factor (eg, serum calcium level) has a causal effect on the health outcomes (eg, osteoporosis), the genetic variants that are associated with the modifiable risk factor should also be associated with the outcome (Fig. 1). The name Mendelian randomization comes from the fact that genetic variants are randomly and independently assigned in a population at conception (following from Mendel's second law); therefore, the potential confounding effects influencing the relationship between the exposure and outcome are generally decreased. Moreover, the assignment of genetic variants during meiosis always happens before the onset of disease, making the bias due to reverse causation improbable. MR has helped to better understand the causal relationships between nutrients and diseases. For example, the protective effect of vitamin D on multiple sclerosis was demonstrated by Mokry and colleagues^(^ ^16^ ^)^ supporting the importance of vitamin D supplementation for people with multiple sclerosis risk.

MR study designs include one‐sample MR (where the effects of genetic variant‐exposure and genetic variant‐outcome are assessed in the same cohort) and two‐sample MR (where the effects of genetic variant‐exposure and genetic variant‐outcome are obtained from two different cohorts). In an MR study, genetic variants are used as instrumental variables if they are associated with the outcome only through their associations with the risk factor. Specifically, the instrumental variables are selected based on three major assumptions (Fig. 1): (i) they are strongly associated with exposure, (ii) they are not associated with confounding factors that affect the relationship between the exposure and outcome, and (iii) they are only associated with the outcome through the exposure. Next, depending on the number of instrumental variables, different statistical methods can be used to estimate the effect of exposure on outcome. For example, the Wald test is often applied when there is only one instrumental variable whereas the inverse‐variance weighting is applied when there is more than one instrumental variable.^(^ ^17^ ^)^

To use MR, the exposure needs to be at least partially inheritable. As mentioned in the previous paragraph, one key assumption of the MR approach is that the genetic variants are robustly associated with the exposure. In this regard, serum calcium level is applicable to MR because it is a moderately inheritable trait. Previous twin studies have shown a heritability between 33% and 78% for serum calcium.^(^ ^18^, ^19^, ^20^ ^)^ More recently, GWASs on serum calcium levels have been done in several large cohorts, and the genetic determinants identified can explain up to 2.6% of serum calcium variance, allowing the use of MR in investigating the health effect of genetically predicted serum calcium.^(^ ^21^, ^22^, ^23^ ^)^ Furthermore, the difference in serum calcium caused by genetic variation is comparable to that caused by diet or supplementation. A standard deviation (SD) increase in genetically derived serum calcium is 0.13 mmol/L or 0.51 mg/dL.^(^ ^24^ ^)^ It has been shown that 1200 mg of calcium given as fortified skim milk can lead to an approximate increase of 0.03 mmol/L in total serum calcium at 3 hours.^(^ ^25^ ^)^ A total of 1000 mg of calcium citrate supplements can lead to 0.10 ± 0.07 mmol/L (total serum calcium) and 0.06 ± 0.03 mmol/L (ionized serum calcium) at 3 hours.^(^ ^26^ ^)^ This evidence supports the use of MR in studying the effect of serum calcium complementary to RCTs.

### Musculoskeletal diseases

Musculoskeletal diseases include diseases that affect bones (eg, osteoporosis and fractures), joints (eg, osteoarthritis and osteoarthrosis), and muscles (eg, sarcopenia). Recent analysis of Global Burden of Disease data shows that musculoskeletal disorders influenced overall 1.7 billion people globally, including 456 million people experiencing fractures and 343 million people having osteoarthritis.^(^ ^27^ ^)^ The increased social and economic burdens of musculoskeletal diseases make their prevention a major public health objective. Calcium and vitamin D supplementation are recommended by health authorities to prevent osteoporosis and fractures. However, controversial findings regarding the benefits of these supplements on reducing bone mineral density (BMD) and fracture risk have been reported in observational studies and RCTs. In a systematic review that analyzed results from 50 observational studies with participants aged >50 years, the authors found that 74% of these studies reported neutral relationship between calcium intake and fracture outcomes, whereas most of the remaining observational studies reported an inverse association.^(^ ^28^ ^)^ In a meta‐analysis that involved 29 RCTs (*n* = 63,897), it was found that the use of calcium or calcium in combination with vitamin D supplementation was associated with a reduced rate of BMD loss and a risk reduction in fractures of all types among people aged >50 years.^(^ ^29^ ^)^ However, a more recent meta‐analysis involving 33 randomized trials (*n* = 51,145) found no significant association of calcium, vitamin D, nor their combination with the risk of hip fracture as compared with placebo or no treatment in community‐dwelling older adults.^(^ ^30^ ^)^

In comparison, MR studies did not find causal relationships between serum calcium level and BMD or fracture risk. Cerani and colleagues^(^ ^24^ ^)^ applied the two‐sample MR method using large GWASs for estimated BMD (eBMD) from heel ultrasound measurements (*n* = 426,824) and fracture risk (76,549 cases and 470,164 controls). They reported that a 1‐SD (0.51 mg/dL) increase in genetically determined serum calcium level was not associated with eBMD nor fracture risk. This finding is in accordance with other MR studies which found that the genetically determined serum calcium levels were not significantly associated with whole‐body BMD^(^ ^31^, ^32^ ^)^ nor the osteoporosis risk.^(^ ^33^ ^)^ Interestingly, these MR studies also found that the higher serum calcium levels had a negative causal effect on BMD (beta: −0.431; *p* = 0.014) after adjusting for the serum PTH, 25(OH)D, and phosphate levels,^(^ ^31^ ^)^ as well as BMD at a specific site such as the lumbar spine (beta: −0.55; *p* = 0.001).^(^ ^32^ ^)^

As for other musculoskeletal diseases, a causal association between higher genetically predicted serum calcium levels and lower risk of osteoarthrosis (odds ratio [OR] 0.67; *p* = 0.0044) was reported in a phenome‐wide MR analysis.^(^ ^34^ ^)^ A two‐sample MR study found that serum calcium levels were also inversely associated with the risk of overall osteoarthritis (OR 0.712; *p* = 1.84 × 10^−4^), although this significant association was only detected in women (OR 0.967; *p* = 0.038).^(^ ^35^ ^)^ Similar findings were found in a recent MR where a negative causal effect of higher serum calcium on localized osteoarthritis was reported (OR 0.87, *p* = 0.021).^(^ ^36^ ^)^ Furthermore, there was no significant causal effect of calcium concentration detected on the risk for rheumatoid arthritis^(^ ^33^, ^36^ ^)^ or gout^(^ ^33^ ^)^ in MR studies.

Together, MR found that higher serum calcium levels do not appear to improve BMD or reduce risk for fracture in the general population. However, they may have some benefits in reducing the risk for osteoarthrosis and osteoarthritis.

### Cardiovascular diseases

Cardiovascular disease (CVD) is the leading cause of death worldwide.^(^ ^37^ ^)^ Cardiac diseases include conditions that affect the heart itself such as atrial fibrillation and heart failure, whereas vascular diseases involve narrowing or blocking of blood vessels in hearts and other tissues such as coronary artery diseases and ischemic stroke. Considering the essential role of calcium in signaling transduction and muscle contraction in heart, many studies have assessed the effect of calcium status on CVD risk. Some observational studies found that higher serum calcium levels increase the risk for coronary artery diseases, in particular myocardial infarction.^(^ ^14^, ^38^ ^)^ Similar positive association was observed between serum calcium and risk for ischemic stroke.^(^ ^38^, ^39^ ^)^ However, inconsistent findings have been reported in observational studies focusing on heart failure. Van Hemelrijck and colleagues^(^ ^40^ ^)^ found no association between heart failure with baseline blood calcium using the data from the third National Health and Nutrition Examination Survey (*n* = 20,024) whereas Lutsey and colleagues^(^ ^41^ ^)^ detected a positive association between serum calcium and heart failure risk using data from an Atherosclerosis Risk in Communities cohort (*n* = 14,709). There were few RCTs that directly investigated the association between calcium supplements and CVD risk. However, several meta‐analyses have used data from calcium intervention RCTs where events of CVD were collected as secondary or adverse outcomes. In a meta‐analysis of RCTs published in 2010, the authors analyzed both patient level data from five trials (*n* = 8151) and trial level data from 11 trials (*n* = 11,921). They found an increased incidence of myocardial infarction in those allocated to the calcium supplementation group.^(^ ^42^ ^)^ The same team conducted a meta‐analysis using patient‐level data from six RCTs (*n* = 24,869) and found similar results that only calcium or calcium with vitamin D supplements increased the risk for myocardial infarction and the composite end point of myocardial infarction or stroke.^(^ ^43^ ^)^ Interestingly, this association was not supported by a meta‐analysis published in 2015 where the authors used data from seven RCTs (*n* = 51,111) and found no increased risk in calcium‐treated patients for myocardial infarction in elderly women.^(^ ^44^ ^)^ A large RCT involving women aged 50 to 79 years (*n* = 35,983) found no association between calcium plus vitamin D supplements and risk of heart failure.^(^ ^45^ ^)^

MR was used to study the role of calcium on various types of cardiovascular diseases. Using the same GWAS (outcome: 60,801 coronary artery disease cases and 123,504 controls), Larsson and colleagues^(^ ^46^ ^)^ and Xu and colleagues^(^ ^47^ ^)^ investigated the causal relationship between serum calcium level and risk for coronary artery disease. Although the selection criteria for instrumental variables were different, both groups found that an increase in genetically predicted serum calcium levels were positively associated with risks for coronary artery disease (Larsson and colleagues^(^ ^46^ ^)^ OR 1.25, *p* = 0.003; Xu and colleagues^(^ ^47^ ^)^ OR 1.49, *p* < 0.05) and myocardial infarction (Larsson and colleagues^(^ ^46^ ^)^ OR 1.24, *p* = 0.009; Xu and colleagues^(^ ^47^ ^)^ OR 1.58, *p* = 0.009). Xu and colleagues^(^ ^47^ ^)^ also found that increased serum calcium levels has an estimated causal effect on previously identified risk factors for CVD such as low‐density lipoprotein (LDL)‐cholesterol (beta 0.21; *p* < 0.05) and total cholesterol (beta: 0.29; *p* < 0.05). The causal effect of serum calcium on myocardial infarction was also investigated by Zhou and colleagues^(^ ^34^ ^)^ using data from UK Biobank (9828 cases and 311,419 controls). They found that higher serum calcium was associated with higher risk for myocardial infarction (OR 1.99; *p* = 0.011). They also reported no causal relationship between serum calcium and metabolic traits such as risk for obesity, lipid metabolic disorders, high‐density lipoprotein (HDL) cholesterol level, LDL cholesterol level, total cholesterol, or total triglyceride levels. As for other subtypes of CVD, no estimated causal effect of genetically predicted serum calcium level was found on atrial fibrillation,^(^ ^48^ ^)^ heart failure,^(^ ^49^ ^)^ or ischemic stroke.^(^ ^50^ ^)^

In summary, converging evidence from RCTs and MR suggested positive causal influence of serum calcium level on risk for coronary artery disease, especially upon myocardial infarction, indicating that calcium supplementation may increase risks for coronary artery diseases. However, MR results did not support any causal relationship between calcium and other cardiovascular conditions such as atrial fibrillation, heart failure, and ischemic stroke.

### Cancer

Cancer is the second leading cause of death worldwide and it is responsible for nearly 10 million deaths per year.^(^ ^51^ ^)^ The top four most commonly diagnosed cancers are lung cancer, breast cancer, prostate cancer, and colorectal cancer, which are also among the leading causes for death due to cancer.^(^ ^51^ ^)^ Because different types of cancers could have distinct pathogenetic mechanisms, it is not surprising to see different effects of calcium status on cancer outcomes. Some studies found that high calcium intake may lower the risk for some common types of cancers such as colorectal cancer, breast cancer, and ovarian cancer. For example, in the American Cancer Society's Cancer Prevention Study II Nutrition Cohort, which included more than 120,000 individuals, higher calcium intake, especially from calcium supplements, was associated with a reduced risk for colorectal cancer.^(^ ^52^ ^)^ Similar observations were found in a combined analysis of the Nurses' Health Study and the Health Professionals Follow‐up Study with more than 135,000 participants.^(^ ^53^ ^)^ A meta‐analysis that included three RCTs (*n* = 1485) found that calcium supplementation was associated with lower recurrence of colorectal adenomas.^(^ ^54^ ^)^ However, contrasting findings were reported by a randomized, double‐blind, placebo‐controlled trial (*n* = 36,282), where they found that 1000‐mg calcium supplements plus vitamin D (400 international units) per day for an average duration of 7 years was not associated with reduced risk of colorectal cancer.^(^ ^55^ ^)^ The Women's Health Study, with more than 30,000 women, found that higher total calcium intake was associated with a reduced risk for breast cancer.^(^ ^56^ ^)^ A meta‐analysis including 15 observational studies also showed a significant inverse relationship between calcium intake and breast cancer incidence, although the effect size became smaller after the publication bias was corrected.^(^ ^57^ ^)^ As for other types of cancer, weak negative associations between dietary calcium intake and the risk for ovarian cancer and astrocytic glioma were reported.^(^ ^58^, ^59^ ^)^

Interestingly, some studies detected positive associations between calcium intake and the risk for certain types of cancer. For example, a meta‐analysis including nine cohort studies (*n* = 750,275) published by Aune and colleagues^(^ ^60^ ^)^ found that higher dietary calcium intake was associated with an increased risk of prostate cancer. Another prospective study involving 27,293 men from Singapore Chinese population also found that higher dietary calcium intake was associated with higher incidence for prostate cancer.^(^ ^61^ ^)^

In general, evidence from MR studies does not support a causal effect of calcium levels on risks for colorectal cancer, breast cancer, or ovarian cancer. Two MR studies using different large meta‐analyses of colorectal cancer GWAS reported that higher serum calcium (1‐SD increase) was not significantly associated deceased risk for colorectal cancer.^(^ ^62^, ^63^ ^)^ An MR study using data from the Breast Cancer Association Consortium (22,977 cases and 105,974 controls) found a suggestive negative association between genetically determined serum calcium and breast cancer risk (OR 0.91; *p* = 0.06), but this finding was not replicated when the authors used instrumental variables from another calcium GWAS.^(^ ^64^ ^)^ In addition, an unclear effect of calcium on invasive epithelial ovarian cancer and low malignant potential tumors was reported in a recent two‐sample MR study.^(^ ^65^ ^)^ Evidence from MR also does not support the positive association between calcium level and risk for prostate cancer. A large‐scale two‐sample MR study (44,825 cases and 27,904 controls) reported a nonsignificant effect of serum calcium concentration on prostate cancer.^(^ ^66^ ^)^ An MR study (12,488 glioma cases and 18,169 controls) found that higher genetically predicted serum calcium levels were estimated to reduce the risk of glioma (OR 0.84; *p* = 0.027); however, this result did not pass the multiple testing correction threshold.^(^ ^67^ ^)^

Overall, evidence from MR does not support the role of serum calcium concentration in influencing cancer risk.

### Neurological diseases

Neurological diseases are the leading cause of disability‐adjusted life‐years globally.^(^ ^68^ ^)^ Because the prevalence of disabling neurological diseases increases with age, the neurological disease‐related social and economic burdens increase as many countries are facing population aging.^(^ ^68^ ^)^ Calcium dysregulation has been found in patients with neurological disorders, and calcium is involved in the pathogenesis of many neurological diseases such as Parkinson disease and Alzheimer disease.^(^ ^69^, ^70^ ^)^ The association between serum calcium levels or calcium intake and Parkinson disease has been evaluated in several observational studies but their findings were inconsistent. For example, Meamar and colleagues^(^ ^71^ ^)^ found that patients with Parkinson disease had lower serum calcium compared to healthy controls. However, in a larger observational study with over 130,000 participants, higher total calcium intake from food was found to be associated with higher risk for Parkinson disease.^(^ ^72^ ^)^ Studies focusing on the association between calcium status and Alzheimer disease also generated inconclusive results. A meta‐analysis published in 2014 retrieved data from four studies that measured calcium level in Alzheimer disease patients.^(^ ^73^ ^)^ Of these four studies, only one found lower calcium levels in Alzheimer disease patients as compared to the controls, whereas others found no difference. A recent observational study focusing on Japanese individuals with mild cognitive impartment (*n* = 234) found that lower serum calcium levels may be associated with an increased risk of the transition from mild cognitive impartment to Alzheimer disease.^(^ ^74^ ^)^ Another observational study also reported that higher calcium intake was associated with lower risk for Alzheimer disease in a Japanese population.^(^ ^75^ ^)^

Cheng and colleagues^(^ ^33^ ^)^ used two‐sample MR to survey the causal relationship between serum calcium and several types of neurological diseases. The authors found that serum calcium levels had no estimated causal effect on risks of Alzheimer disease, bipolar disorder, schizophrenia, Parkinson disease, and major depressive disorder.^(^ ^33^ ^)^ For Parkinson disease, another two‐sample MR study also reported no direct causal effect of calcium on the disease risk.^(^ ^76^ ^)^ Surprisingly, using the same exposure and outcome GWAS as Cheng and colleagues,^(^ ^33^ ^)^ He and colleagues^(^ ^77^ ^)^ reported a significantly negative causal effect between serum calcium concentration and risk of Alzheimer disease (OR 0.57; *p* =  0.031). This was likely because these two studies implemented different variant selection and proxy identification criteria when selecting instrumental variables. MR was also used to investigate the association between serum calcium and other neurological disorders. Yuan and colleagues^(^ ^78^ ^)^ found that there was no significant causal relationship between calcium levels and risk for epilepsy. Another two‐sample MR study (23,285 migraine cases and 95,425 controls) found that serum calcium levels were significantly associated with higher risk for migraine (OR 1.80; *p* = 2.5 × 10^−4^).^(^ ^79^ ^)^

Overall, higher serum calcium levels are unlikely to reduce the risk for many neurological diseases, and may increase the risk for migraine.

### Diabetes

Calcium is involved in insulin secretion and action. Altered calcium homeostasis plays a role in the glucose metabolism and the development of diabetes, especially type 2 diabetes (T2D).^(^ ^80^ ^)^ Inconsistent findings have been reported on the effect of calcium on diabetes risk in different studies. A prospective study (*n* = 64,191) found that moderate calcium supplementation was associated lower risk for T2D in Chinese women.^(^ ^81^ ^)^ However, a recent retrospective cohort study with 6096 Hong Kong Chinese participants found that higher baseline total calcium was associated with higher risk for diabetes.^(^ ^82^ ^)^ They also conducted a meta‐analysis of four observational studies including the prospective study mentioned above and found that higher serum total calcium levels were associated with higher relative risk for diabetes.

MR methods were used to investigate the causal effect of calcium level on the risks of type 2 diabetes. Yuan and colleagues^(^ ^83^ ^)^ used combined data from 32 studies (74,124 cases and 824,006 controls) in a two‐sample MR analysis, and found that the genetically predicted calcium was not associated with risk for T2D. Similar null results were reported by another group using different T2D meta‐analysis GWAS data (62,892 cases and 596,424 control) and found serum calcium level had no effect on T2D risk.^(^ ^33^ ^)^

### Other traits

Some MR studies also explored the potential causal relationship between calcium and other traits. Thompson and colleagues^(^ ^84^ ^)^ used two‐sample MR to investigate the association between maternal serum calcium and offspring birth weight because several observational studies found that the low maternal calcium level was associated with low infant birth weight.^(^ ^85^, ^86^ ^)^ A systematic review of available calcium supplementation RCTs at the time also concluded that calcium supplementation can increase infant birth weight.^(^ ^87^ ^)^ However, using GWAS data from 190,406 women and the birth weight of their first child, Thompson and colleagues^(^ ^84^ ^)^ found that genetically predicted high maternal serum calcium level had no estimated causal effect on offspring birth weight.

Finally, using two‐sample MR method, Zhou and colleagues^(^ ^34^ ^)^ found high serum calcium concentration has an estimated causal effect on urinary calculus (OR 3.5; *p* = 0.011), renal colic (OR 9.1; *p* = 8.82 × 10^−4^), and allergy/adverse effect of penicillin (OR 2.2; *p* = 9.36 × 10^−5^).

## Overall Summary and Conclusion

Overall, the MR studies found no estimated causal effect of serum calcium levels on most of the health outcomes tested. Specifically, available MR studies showed no direct causal effect of serum calcium level on improving BMD or reducing fracture risk, although higher serum calcium concentration may reduce the risk for osteoarthrosis and osteoarthritis. High calcium levels were found to have an estimated causal effect on the higher risk for coronary artery disease (ie, myocardial infarction), but not heart failure, atrial fibrillation, and ischemic stroke. No estimated causal effect was detected between serum calcium status and the risk for common cancers. No conclusive relationships were found between serum calcium status and risk for certain chronic diseases (ie, gout, rheumatoid arthritis, and type 2 diabetes), neurological diseases (ie, Alzheimer disease, bipolar disorder, schizophrenia, Parkinson disease, and major depressive disorder), or birth weight. However, higher levels of serum calcium had an estimated causal effect on increased risks for migraine, urinary calculus, renal colic, and allergy/adverse effect of penicillin (Table 1).

**Table 1:** Calcium and Different Health Outcomes: Evidence from Mendelian Randomization Studies

Study	Number of genetic instruments	Number of participants in outcome GWAS (cases/controls)	Outcome	Estimate of effect OR/Beta (95% CI)	*p*	Unit of estimated effect
Cerani and colleagues^(^ ^24^ ^)^ (2019)	6	426,824	eBMD	0.0003 (−0.059, 0.066)	0.92	1 SD (0.51 mg/dL)
	6	76,549/470,164	Fracture risk	1.01 (0.89, 1.15)	0.85	1 SD (0.51 mg/dL)
Li and colleagues^(^ ^31^ ^)^ (2020)	7	66,628	BMD	−0.139 (−0.290, 0.012)	0.072	1 SD (0.35 mg/dL)
Qu and colleagues^(^ ^32^ ^)^ (2021)	7	66,628	BMD	−0.4 (−0.83, 0.04)	0.072	1 SD
	7	28,498	Lumbar spine BMD	−0.55 (−0.86, −0.24)	0.001	1 SD
Cheng and colleagues^(^ ^33^ ^)^ (2019)	6	98,742/409,511	Osteoporotic fractures	0.39 (0.15, 1.07)	0.068	1 SD (0.55 mg/dL)
	7	2115/67,259	Gout	2.84 (0.45, 17.92)	0.267	1 SD (0.55 mg/dL)
	6	29,880/73,758	Rheumatoid arthritis	1.83 (0.99, 3.41)	0.055	1 SD (0.55 mg/dL)
Zhou and colleagues^(^ ^34^ ^)^ (2019)	7	36,434/301,101	Osteoarthrosis	0.67 (0.51, 0.88)	0.0044	1 mg/dL
Qu and colleagues^(^ ^35^ ^)^ (2020)	7	30,046/331,095	Osteoarthritis	0.712 (0.595, 0.850)	0.000184	NA
Zhou and colleagues^(^ ^36^ ^)^ (2021)	6	36,612/274,387	Osteoarthritis	0.87 (0.76, 1.01)	0.06	1 SD
	6	30,741/280,258	Localized osteoarthritis	0.83 (0.69, 0.98)	0.021	1 SD
	6	2411/286,995	Rheumatoid arthritis	0.73 (0.46, 1.14)	0.16	1 SD
Larsson and colleagues^(^ ^46^ ^)^ (2017)	6	60,801/123,504	Coronary artery disease	1.25 (1.08, 1.45)	0.003	1 SD (0.5 mg/dL)
Xu and colleagues^(^ ^47^ ^)^ (2017)	4	60,801/123,504	Coronary artery disease	1.49 (1.02, 2.17)	<0.05	1 mg/dL
Zhou and colleagues^(^ ^34^ ^)^ (2019)	7	9828/311,419	Myocardial infarction	1.99 (1.17, 3.39)	0.011	1 mg/dL
	7	43,676/128,199	Myocardial infarction	1.48 (1.08, 2.02)	0.015	1 mg/dL
Larsson and colleagues^(^ ^48^ ^)^ (2019)	7	65,446/522,744	Atrial fibrillation	1.08 (0.96, 1.21)	0.20	1 SD
Helte and colleagues^(^ ^49^ ^)^ (2019)	7	6504/387,652	Heart failure	0.89 (0.67, 1.17)	0.41	1 SD (0.5 mg/dL)
Larsson and colleagues^(^ ^50^ ^)^ (2019)	7	34,217/404,630	Ischemic stroke	1.03 (0.88, 1.21)	0.68	1 SD (0.5 mg/dL)
Cornish and colleagues^(^ ^62^ ^)^ (2020)	8	26,397/41,481	Colorectal cancer	0.93 (0.83, 1.05)	0.26	1 SD
Tsilidis and colleagues^(^ ^63^ ^)^ (2021)	7	58,221/67,694	Colorectal cancer	0.85 (0.74, 0.96)	0.01^a^	1 SD (0.48 mg/dL)
	207	58,221/67,694	Colorectal cancer	1.02 (0.95, 1.11)	0.55	1 SD (0.48 mg/dL)
Papadimitriou and colleagues^(^ ^64^ ^)^ (2021)	7	122,977/105,974	Breast cancer	0.91 (0.83, 1.00)	0.06	1 SD (0.48 mg/dL)
Guo and colleagues^(^ ^65^ ^)^ (2020)	7	22,406/40,941	Epithelial ovarian cancer	1.24 (0.78, 1.98)	0.36	1 SD (0.5 mg/dL)
Yarmolinsky and colleagues^(^ ^66^ ^)^ (2018)	5	44,825/27,904	Prostate cancer	0.83 (0.63, 1.08)	0.12^b^	1 SD (0.5 mg/dL)
Saunders and colleagues^(^ ^67^ ^)^ (2019)	8	12,488/18,169	Glioma	0.84 (0.71, 0.98)	0.0278^a^	1 SD
Wang and colleagues^(^ ^76^ ^)^ (2020)	6	4238/4239	Parkinson disease	1.73 (0.47, 6.37)	0.408	1 SD (0.5 mg/dL)
	6	4627/109,826	Parkinson disease	1.39 (0.45, 4.31)	0.564	1 SD (0.5 mg/dL)
He and colleagues^(^ ^77^ ^)^ (2020)	6	17,008/37,154	Alzheimer disease	0.57 (0.35, 0.95)	0.031	1 SD (0.5 mg/dL)
Cheng and colleagues^(^ ^33^ ^)^ (2019)	7	4238/4239	Parkinson disease	1.57 (0.49, 5.02)	0.44	1 SD (0.55 mg/dL)
	6	17,008/37,154	Alzheimer disease	0.74 (0.45, 1.22)	0.23	1 SD (0.55 mg/dL)
	7	20,129/21,524	Bipolar disorder	1.85 (0.74, 4.65)	0.19	1 SD (0.55 mg/dL)
	7	33,426/32,541	Schizophrenia	0.81 (0.53, 1.23)	0.32	1 SD (0.55 mg/dL)
Yuan and colleagues^(^ ^78^ ^)^ (2021)	6	4588/144,780	Epilepsy	0.93 (0.7, 1.24)	0.62	1 SD
Yin and colleagues^(^ ^79^ ^)^ (2016)	8	23,285/95,425	Migraine	1.80 (1.31, 2.46)	0.00025	1 mg/dL
Cheng and colleagues^(^ ^33^ ^)^ (2019)	7	62,892/596,424	Type 2 diabetes	0.88 (0.34, 2.27)	0.80	1 SD (0.55 mg/dL)
Yuan and colleagues^(^ ^83^ ^)^ (2019)	6	74,124/824,006	Type 2 diabetes	1.05 (0.93, 1.18)	0.462	1 SD (0.5 mg/dL)
Thompson and colleagues^(^ ^84^ ^)^ (2019)	7	190,406	Birth weight	−20.00 (−44.00, 5.00)	0.116	1 SD (0.5 mg/dL)
Zhou and colleagues^(^ ^34^ ^)^ (2019)	7	5641/330,763	Urinary calculus	3.51 (1.34, 9.17)	0.011	1 mg/dL
	7	1689/33,0763	Renal colic	9.11 (2.48, 33.5)	0.000882	1 mg/dL
	7	13,152/31,3986	Allergy/adverse effect of penicillin	2.21 (1.49, 3.30)	0.0000936	1 mg/dL

For the studies that applied multiple MR methods, results from inverse‐variance weighted estimation are shown.

CI = confidence interval.

^a^ The corresponding study tested more than one outcome and the *p* value did not pass the multiple testing correction threshold set by the authors.

^b^ Likelihood‐based method to combine Wald test estimates.

Although MR can address the bias of observational studies and RCTs, it has its own limitations. First, the quality and utility of MR studies are dependent on the quality and nature of the GWAS. Using summary statistics from a GWAS with small sample size reduces the power of MR in detecting small to modest effects, leading to null findings.^(^ ^88^ ^)^ In addition, all MR studies discussed in this review used GWAS with a majority of participants from European ancestry, and consequently results may not be applicable in other ethnic groups. Second, the selection of instrumental variables has an important impact on the results. We have observed that different selection criteria of instrumental variables leads to different results even when the exposure and outcome GWAS are the same across studies. Third, many MR studies are unable to capture the causal relationship in specific demographic groups because the subpopulation GWAS are less available. Most importantly, nearly all of the MR studies reported here assess the linear relationship between circulating calcium levels and disease outcomes. As with vitamin D and rickets, these relationships may be nonlinear. However, the current MR literature gives a reasonable estimation of the effect of increasing calcium supplementation in the general population. MR results can be influenced by canalization which is defined as a compensatory developmental process induced by the disruptive effect of the genetic variation.^(^ ^89^ ^)^ This usually cannot be adjusted in the MR analysis. Fortunately, many of these limitations could be soon addressable as more large‐scale GWASs from deeply phenotyped biobanks are becoming increasingly available. This could help to improve the quality of MR by providing stronger genetic instruments and more data of populations with diverse ancestral backgrounds, adding power to the causal effect estimation and generalizability.

In conclusion, available MR studies did not observe estimated causal effects of serum calcium level on many health outcomes. Increasing serum calcium levels in the general population may provide little health benefits, and on the contrary, could impose higher risks for certain medical conditions like coronary artery disease, renal colic, and migraine. Supplementation of calcium in non–calcium‐deficient individuals is unlikely to provide additional benefits. Instead, long‐term high serum calcium concentration may increase risk for certain diseases. Future studies should assess potential nonlinear relationships between serum calcium and disease outcomes.

## Conflict of Interest

JBR has served as an advisor to GlaxoSmithKline and Deerfield Capital. His institution has received investigator‐initiated grant funding from Eli Lilly, GlaxoSmithKline and Biogen for projects unrelated to this research. He is the founder of 5 Prime Sciences. YC, VF, and SZ declare no conflict of interest.

### Peer Review

The peer review history for this article is available at https://publons.com/publon/10.1002/jbm4.10542.
